# Implementation of anesthesia quality indicators in Germany: A prospective, national, multicenter quality improvement study

**DOI:** 10.1007/s00101-020-00773-y

**Published:** 2020-07-06

**Authors:** S. Ziemann, M. Coburn, R. Rossaint, J. Van Waesberghe, H. Bürkle, M. Fries, M. Henrich, D. Henzler, T. Iber, J. Karst, O. Kunitz, R. Löb, W. Meißner, P. Meybohm, B. Mierke, F. Pabst, G. Schaelte, J. Schiff, M. Soehle, M. Winterhalter, A. Kowark

**Affiliations:** 1 Department of Anaesthesiology, Medical Faculty, RWTH Aachen University, Pauwelsstr. 30, 52074 Aachen, Germany; 2 Department of Anaesthesiology and Critical Care Medicine, Faculty of Medicine, University Hospital Freiburg, Freiburg, Germany; 3 Department of Anaesthesiology, St. Vincenz Hospital Limburg, Limburg, Germany; 4 Department of Anaesthesiology and Critical Care Medicine, St.-Vincentius Hospital Karlsruhe, Karlsruhe, Germany; 5 Department of Anaesthesiology, Surgical Intensive Care, Emergency and Pain Medicine, Klinikum Herford, Ruhr-University Bochum, Herford, Germany; 6 Department of Anaesthesiology, Critical Care and Pain Medicine, Klinikum Mittelbaden, Baden-Baden, Germany; 7 Outpatient Anaesthesia Care Centre Karst, Berlin, Germany; 8 Department of Anaesthesiology and Critical Care Medicine, Klinikum Mutterhaus der Borromäerinnen, Trier, Germany; 9 Department of Anaesthesiology, Critical Care, Emergency and Pain Medicine, St. Barbara Hospital, Hamm, Germany; 10 Department of Anaesthesiology and Critical Care Medicine, University Hospital Jena, Jena, Germany; 11 Department of Anaesthesiology, Intensive Care Medicine and Pain Therapy, University Hospital Würzburg, Würzburg, Germany; 12 Department of Anaesthesiology and Critical Care Medicine, Hospital St. Elisabeth, Damme, Germany; 13 Department of Anaesthesiology and Critical Care Medicine, University Hospital Rostock, Rostock, Germany; 14 Department of Anaesthesiology and Surgical Intensive Care Medicine, Klinikum Stuttgart, Stuttgart, Germany; 15 Department of Anaesthesiology and Critical Care Medicine, University Hospital Bonn, Bonn, Germany; 16 Department of Anaesthesiology and Pain Medicine, Klinikum Bremen-Mitte, Bremen, Germany

**Keywords:** Anaesthesia, Quality indicators, Quality of Healthcare, Benchmarking, Quality assurance, Anästhesie, Qualitätsindikatoren, Qualität der Gesundheitsversorgung, Benchmarking, Qualitätssicherung

## Abstract

**Background:**

In 2016 the German Society of Anesthesiology and Intensive Care Medicine (DGAI) and the Association of German Anesthetists (BDA) published 10 quality indicators (QI) to compare and improve the quality of anesthesia care in Germany. So far, there is no evidence for the feasibility of implementation of these QI in hospitals.

**Objective:**

This study tested the hypothesis that the implementation of the 10 QI is feasible in German hospitals.

**Material and methods:**

This prospective three-phase national multicenter quality improvement study was conducted in 15 German hospitals and 1 outpatient anesthesia center from March 2017 to February 2018. The trial consisted of an initial evaluation of pre-existing structures and processes by the heads of the participating anesthesia departments, followed by a 6-month implementation phase of the QI as well as a final re-evaluation phase. The implementation procedure was supported by web-based implementation aids (www.qi-an.org) and internal quality management programs. The primary endpoint was the difference in the number of implemented QI per center before and after implementation. Secondary endpoints were the number of newly implemented QI per center, the overall number of successful implementations of each QI, the identification of problems during the implementation as well as the kind of impediments preventing the QI implementation.

**Results:**

The average number of implemented QI increased from 5.8 to 6.8 (mean of the differences 1.1 ± 1.3; *P* < 0.01). Most frequently the QI *perioperative morbidity* and mortality report (5 centers) and the QI *temperature management* (4 centers) could be implemented. After the implementation phase, the QI *incidence management* and *patient blood management* were implemented in all 16 centers. Implementation of other quality indicators failed mainly due to a lack of time and lack of structural resources.

**Conclusion:**

In this study the implementation of QI was proven to be mostly feasible in the participating German hospitals. Although several QI could be implemented with minor effort, more time, financial and structural resources would be required for some QI, such as the QI postoperative visit.

## Abstract (de)

**Hintergrund:**

Im Jahr 2016 hat die Deutsche Gesellschaft für Anästhesiologie und Intensivmedizin (DGAI) und der Bund Deutscher Anästhesisten (BDA) 10 Qualitätsindikatoren (QI) veröffentlicht, um die Qualität anästhesiologischer Behandlungen vergleichen und verbessern zu können. Bis heute gibt es keine Evidenz darüber, inwieweit sich die Maßnahmen zur Implementierung der QI in den Kliniken umsetzen lassen.

**Ziel der Arbeit:**

Im Rahmen dieser Studie wurde die Hypothese getestet, inwieweit die vorliegenden 10 QI in deutschen Krankenhäusern implementierbar sind.

**Material und Methoden:**

Diese prospektive, dreiphasige, nationale, Multizenterstudie wurde in 15 deutschen Krankenhäusern und einem ambulanten Anästhesie-Zentrum von März 2017 bis Februar 2018 durchgeführt. Die Studie bestand aus einer initialen Evaluation präexistierender Strukturen und Prozesse durch die Chefärzte der beteiligten Anästhesieabteilungen, gefolgt von einer sechsmonatigen Implementierungsphase der QI und einer finalen Reevaluation. Der Prozess der Implementierung wurde durch web-basierte Implementierungshilfen (www.qi-an.org) und interne Qualitätsmanagementprogramme der Zentren unterstützt. Der primäre Endpunkt war die Differenz der Anzahl implementierter QI pro Zentrum vor und nach der sechsmonatigen Implementierungsphase. Sekundäre Endpunkte waren die Anzahl neu implementierter QI pro Zentrum, die Gesamtzahl erfolgreicher Implementierungen pro einzelnem QI, die Identifizierung von Problemen während der Implementierung sowie die Gründe, die die Implementierung eines QI verhindert haben.

**Ergebnisse:**

Die durchschnittliche Anzahl implementierter QI pro Zentrum stieg von 5,8 auf 6,8 (Mittelwert der Differenzen 1,1 ± 1,3; *p* < 0,01). Am häufigsten konnten die QI *Perioperativer Morbiditäts- und Mortalitätsbericht *(5 Zentren) und QI *Temperatur-Management *(4 Zentren) implementiert werden. Nach der Implementierungsphase waren die QI *Fehlermanagement *und *Patient-Blood-Management *in allen Zentren implementiert. Die Implementierung anderer QI scheiterte in den häufigsten Fällen an Zeitmangel sowie fehlenden personellen und strukturellen Ressourcen.

**Diskussion:**

Im Rahmen der Studie konnte die Implementierbarkeit der vorliegenden 10 QI in deutschen Krankenhäusern überwiegend belegt werden. Viele der QI konnten mit relativ geringem Aufwand implementiert werden. Für einige QI wären jedoch mehr Personal sowie größere strukturelle und finanzielle Ressourcen notwendig, so z.B. für den QI *Postoperative Visite.*

Measuring the quality of anesthesia is a vital but challenging task. Therefore, the German Society of Anaesthesiology and Intensive Care Medicine (DGAI) and the Association of German Anaesthetists (BDA) took the first step by publishing 10 quality indicators (QI) in 2016. The present trial was conducted to ascertain the feasibility of these QI to be implemented in German hospitals in clinical routine.

## Introduction

The quality of anesthesia is affected by an array of factors. These include patient-centered variables, such as patient safety and satisfaction, mortality and functional outcome as well as economical aspects, optimized procedural organization and structured ambient conditions along with qualified and motivated medical personnel. These factors can be subsumed into three categories: first, the quality of the institution’s structure, second, the quality of the anesthesia process itself and third, the quality of the outcome and its measurability [5]. Particularly in anesthesia, the discrimination between surgery-related and anesthesia-related criteria is of crucial importance since many outcome-related parameters depend on the success of both disciplines [6]. To compare and improve the quality of anesthesia care a measurable catalogue of relevant criteria is important. The development of quality indicators (QI), initially used in manufacturing processes is a feasible way to condense required information in order to achieve reliable and comparable data, also in healthcare [8]. Therefore, the executive committees of the German Society of Anaesthesiology and Intensive Care Medicine (DGAI) and the Association of German Anaesthetists (BDA) founded a task force to develop and evaluate QI for anesthesia in Germany. A decision-making process, consisting of an initial literature search followed by three consecutive surveys using the Delphi method among the task force members, took place to elaborate potential QI. Finally, a list of 10 QI was consented by the DGAI and BDA and published in 2016 [5]. Although improvement of patient outcome by enhanced QI adherence is still lacking evidence in any medical subject area, several of the QI are linked to direct impact on patient safety and outcome [3]. This refers, among others, to patient blood management, temperature management and the usage of the WHO (World Health Organization) safe surgery checklist [7, 9, 12]. In this prospective, national, multicenter quality improvement trial, the feasibility of implementation of the 10 QI of the DGAI in 16 German centers after a 6-month implementation phase was investigated. The primary endpoint was the difference in number of implemented QI per center after the intervention compared with the initial assessment.

## Material and methods

**Fig. 1:**
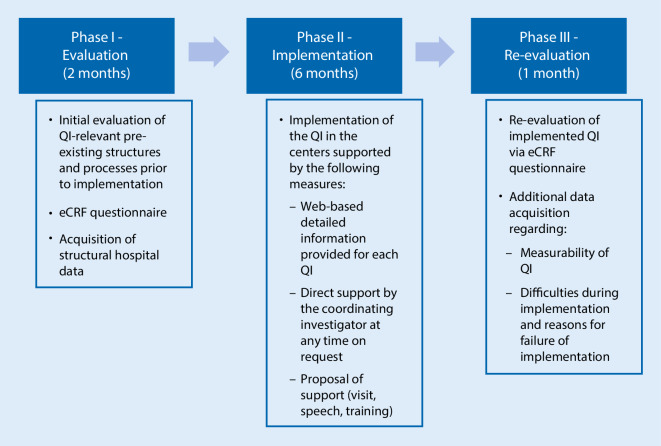
Flowchart of study. Study design with initial evaluation (phase I), implementation (phase II) and final re-evaluation (phase III). *QI* quality indicators, *eCRF* electronic case report form

A total of 20 German centers registered for participation in this prospective, multicenter, quasi-experimental trial. Of the centers 16 successfully completed the entire three phases of the trial, while 4 retracted their offer to participate. Phase I comprised an initial self-evaluation of potentially pre-existing adherence to the QI prior to the QI implementation phase (Fig. 1). Therefore, the participants were asked to report the existence of every single item required for one of the QI. In addition, the center’s structural hospital data regarding anesthesia were acquired. For this purpose, each center was able to access an electronic case report form (eCRF) from March to April 2017, which was set up with an OpenClinica database (OpenClinica, LLC, Waltham, MA, USA). In phase II, the implementation of the 10 QI took place in all participating centers during a 6-month period between May and October 2017 (Fig. 1). For support, the website www.qi-an.org, hosted by Docs in Clouds GmbH, Aachen, Germany, was launched. It provided detailed information regarding each QI, supporting literature as well as helpful tools for their implementation, e.g. presentation slides for employee training. Furthermore, direct contact with the coordinating investigator was possible at any time. A supplemental support by on-site lecture or training was proposed to each center by the coordinating investigator. The particular measures to implement the QI (e.g. teaching, structural improvements) were left to the discretion of each center’s responsibility. For example, the following measures were taken to implement the QI in one of the centers: all missing standard operation procedures (SOP) were elaborated by selected consultants of the department of anesthesiology and implemented after internal revision to meet the criteria of QI I. Implementation of QI II failed, since the anesthesia protocol used by the center is standardized for participation in the Perioperative Medicine Network (PoMNet) which would interfere with the DGAI recommendations. The implementation is likely to be feasible when the centre launches an electronic anesthesia record. Incident management (QI III) was already implemented and did not require further measures. Patient blood management (QI IV) was also already implemented, but the center introduced a system for electronic documentation of indications for transfusion of blood products. Regarding QI V, the centre elaborated a SOP for temperature management. An internal audit of 50 consecutive anesthesia protocols and patient protocols was conducted by a consultant. The aim was to determine whether intraoperative body temperature was measured and the corresponding temperature value at the end of the surgery as well as completion rates required by QI VI (safe surgery checklist) and VII (morbidity and mortality report). As a result, the implementation of QI V and VI failed due to an insufficient documentation rate in the analyzed sample. Items regarding QI VII were completed sufficiently, but not all items could be queried due to PoMNet participation as mentioned. Implementation of QI VIII (handover and discharge protocols) was not possible because of an interfering research project on this topic. Postoperative visits and physician staffing as recommended by DGAI, were already established. Therefore, the implementation of both QI IX as well as QI X was met without additional efforts in this center.

In phase III, following the implementation period, the participating center’s adherence to the QI was re-evaluated by a second self-assessment via eCRF using the same questions as in phase I (Fig. 1). In addition, two supplemental questions were asked of each QI: First, regarding difficulties during the implementation process that might be solved on site. Second, about the causes of implementation failure. Furthermore, the centers were asked to provide their appraisal of whether all necessary data for the QI were registered and accessible in their clinical routines. This phase III assessment took place in February 2018.

Each QI consisted of a different number of items, which were predefined by the QI task force [5]. For each QI comprising more than one item, an individual threshold was defined in order to discriminate whether adherence to a sufficient number of items for the QI could be classified as implemented. For example, for fulfilment of the criteria of QI VI, it was necessary to have both the filed WHO safe surgery checklist in the patient record as a routine procedure as well as a completion rate of this checklist of at least 95%. For a detailed description of all QI including the benchmark criteria, please refer to Table 1. Successful implementation was rated as one point. A maximum sum of ten points was achievable indicating complete adherence to all QI. Fulfilment of routine data from anesthesia records was checked in each center either from a sample of 50 consecutive paper-based records from the preceding month or from the center’s patient data management systems (PDMS). Procedures were left to the discretion of the centers as part of their routine quality assurance. The adherence to the QI in phase I and III was calculated by the investigators according to the participant’s entries in the database. The accuracy of these entries has not been validated.

**Table 1:** Detailed overview of the 10 quality indicators (QI) consented and published by the German Society of Anesthesiology and Intensive Care Medicine (DGAI) and the Association of German Anesthetists (BDA) [5]. Translated and adapted with permission of the authors

**QI I**	**Safety protocols according to the Declaration of Helsinki**	
*Data source*	Safety protocols/standard operation procedures (SOP)	
*Sample*	Availability of all safety protocols according to the Helsinki Declaration:	
	– Checking equipment and drugs	– Preoperative assessment and preparation
	– Syringe labelling	– Massive hemorrhage
	– Postoperative care including pain relief	– Infection control and prevention
	– Anaphylaxis	– Malignant hyperpyrexia
	– Local anesthetic toxicity	– Difficult airway/failed intubation
*Benchmark*	7 out of 10 items: yes/no	
**QI II**	**DGAI’s core dataset 3.X compatible anesthesia record**	
*Data source*	Anesthesia record	
*Sample*	All 66 items of the DGAI’S anesthesia core dataset 3.x need to be included in the local anesthesia record	
*Benchmark*	Yes/no	
**QI III**	**Incident management system**	
*Data source*	Minutes of critical incident reporting system (CIRS) and/or morbidity and mortality (M&M) conference and/or documented case report	
*Sample*	All of the above	
*Benchmark*	At least 4min of CIRS and/or M&M and/or documented case reports per year (two per half-year): yes/no	
**QI IV**	**Patient blood management (PBM)**	
*Data source*	Presence of measures included in PBM at the institution:	
	– Preoperative anemia diagnostic and therapy	– SOP PBM
	– Preoperative coagulation assessment	– SOP transfusion of blood products
	– Hemotherapy algorithm	– SOP massive transfusion (e.g. postpartum haemorrhage, trauma)
	– Measures to reduce diagnostic blood loss	– Documentation of indications for blood transfusion
	– Periodic PBM/hemotherapy education	– Regular reporting (e.g. incidence of anemia, consumption of blood products)
*Benchmark*	2 out of 10 items: yes/no	
**QI V**	**Temperature management**	
*Data source*	Anesthesia record/patient data management system (PDMS): audit of 50 consecutive records	
	Interview of head of department	
*Sample*	– Documented intraoperative temperature recording in >80% of audited interventions >½ h	
	– Core body temperature >36 °C at end of surgery in >70% of audited interventions	
	– SOP temperature management available	
*Benchmark*	3 out of 3 items: yes/no	
**QI VI**	**Safe surgery checklist according to the World Health Organization (WHO)**	
*Data source*	Interview of head of department	
	Patient protocol: audit of 50 consecutive protocols	
*Sample*	– WHO safe surgery checklist in patient protocol by default	
	– WHO safe surgery checklist completed in >95% of sample	
*Benchmark*	2 out of 2 items: yes/no	
**QI VII**	**Perioperative morbidity and mortality report**	
*Data source*	Interview of head of department	
	Anesthesia record: audit of 50 consecutive records	
*Sample*	Presence of item querying:	
	– Mortality (24 h postoperative)	– Visual analogue scale (VAS) >3 when discharged from post anesthetic care unit (PACU)
	– Aspiration	– Postoperative nausea and vomiting (PONV)
	– Puncture-related lesion	
	– Awareness	
	– Patient positioning injury	
	– Items completed in >95% of sample yes/no	
*Benchmark*	8 out of 8 items: yes/no	
**QI VIII**	**Handover and discharge protocols**	
*Data source*	Interview of head of department	
	Anesthesia record/patient protocol: audit of 50 consecutive records/protocols	
*Sample*	– SOP/instruction for handover and discharge protocol in clinical routine	
	– Items completed in >95% of sample	
*Benchmark*	2 out of 2 items: yes/no	
**QI IX**	**Postoperative anesthesiologic visit**	
*Data source*	Interview of head of department	
*Sample*	SOP/instruction for postoperative visit in clinical routine	
*Benchmark*	Standard: yes/no	
**QI X**	**Physician staffing according to DGAI recommendations**	
*Data source*	Interview of head of department	
*Sample*	– Ratio anesthetist:patient 1:1 = 100%; yes/no	
	– Ratio supervisor:junior resident in the first 3 months of anesthesia training 1:1 or 1:2 = 100%; yes/no	
*Benchmark*	2 out of 2 items: yes/no	

The primary endpoint was the difference in the number of implemented QI per center after the implementation phase compared with the initial assessment (phase I). Secondary endpoints were the number of newly implemented QI per center, the overall number of successful implementations of each QI, the identification of problems during the implementation and the kind of impediments preventing the QI implementation, as well as the availability of the required information data from the hospitals’ routine databases. A problem during implementation was defined as a difficulty that could be solved resulting in QI adherence. It could take, for example, 3 months and several meetings before an additional item querying postoperative nausea and vomiting (PONV) was added to a center’s PDMS. In contrast, an impediment was defined as an issue that resulted in termination of a center’s effort to implement the affected QI. As an example, the IT department could refuse the installation of an additional item because of technical barriers. Furthermore, each center’s baseline hospital data were acquired, including the type of institution (university, primary, secondary, tertiary hospital or outpatient center), number of beds, total number of annual anesthesia procedures, hospital’s annual average case mix index and number of personnel, subdivided into residents and specialists. Statistical analysis was performed using Graphpad Prism 7, Graphpad Software, San Diego, CA, USA. The two groups were compared by two-tailed, paired t‑test after verifying Gaussian distribution. An ethical approval was not necessary according to German law. A waiver of the Ethics Committee of the Medical Faculty, RWTH Aachen University, Aachen, Germany (Chairperson Prof G. Schmalzing) was obtained (EK 071/19). This manuscript was written according to the SQUIRE 2.0 guidelines [15].

## Results

### Baseline hospital data

Among the 16 centers there were 7 university hospitals, 6 primary hospitals, 1 secondary and 1 tertiary hospital as well as 1 outpatient anesthesia centre. The capacity of the hospitals (excluding the outpatient centre) ranged from 244 to 1610 beds (mean 912). The number of anesthesia procedures per year ranged from 6041 to 33,649 (mean 17,803). The annual average case mix index ranged from 0.9 to 1.9 (mean 1.2). The number of anesthesia physicians ranged from 14 to 146 (mean 65.2), among them were 3–72 residents (mean 31.8) and 6–74 specialists (mean 33.8).

### QI implementation

**Fig. 2:**
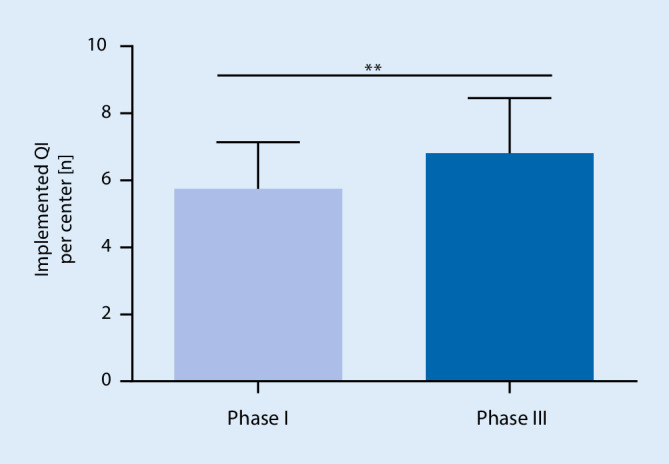
Primary endpoint. Average number of implemented quality indicators (QI) per center before (phase I) and after (phase III) the study intervention, mean absolute difference 1.1 (SD 1.3; 95% CI 0.3–1.8; ** *p* < 0.01 in two-tailed, paired t‑test)

**Fig. 3:**
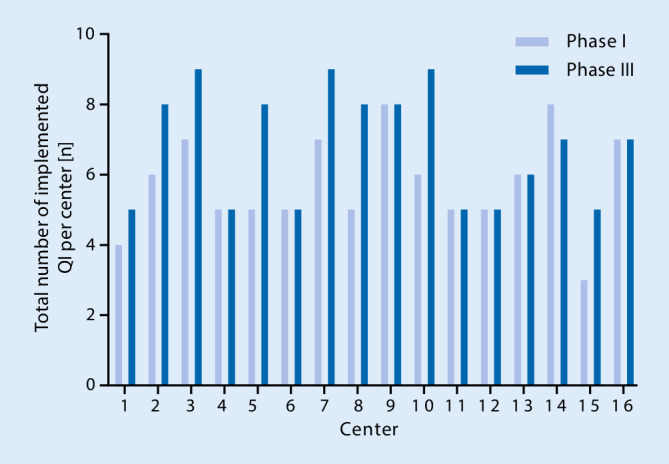
Implemented quality indicators (QI) per hospital. Overview of the individual success of implementation in each study center shown as the number of implemented QI prior to and after study intervention

**Fig. 4:**
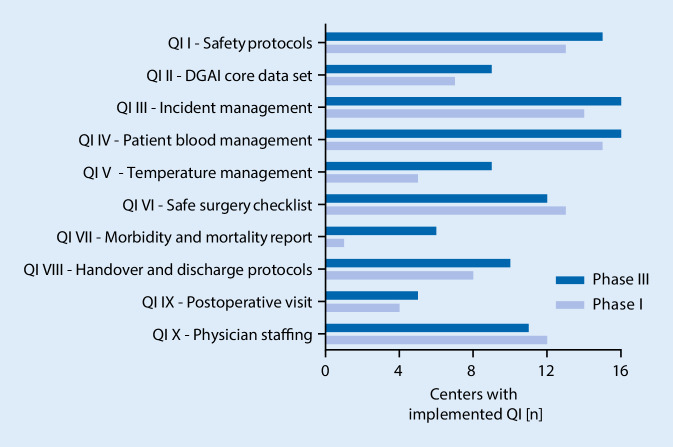
Number of implementations for each quality indicator (QI). Number of centers having implemented each individual QI prior to and after study intervention

The average number of implemented QI increased from 5.8 to 6.8 in total (mean of differences 1.1 ± 1.3; 95% confidence interval, CI 0.3–1.8; *P* < 0.01) (Fig. 2). The range of implemented QI was 3–8 before and 5–9 after the study intervention phase. A maximum of three new QI could be implemented per center (Fig. 3). The most frequently newly implemented QI were QI VII*—perioperative morbidity and mortality report* (five centers) and QI V*—temperature management* (four centers). Conversely, the criteria for QI VI*—WHO safe surgery checklist* and QI X*—physician staffing according to DGAI recommendations* were less frequently fulfilled after study completion (Fig. 4). Of note, the figures mask the fact that one center could implement the QI VI, whereas two centers lost their adherence to the QI criteria in the course of the study. In phase III, all centers have successfully implemented the QI III*—incident management system* and the QI IV*—patient blood management*. In contrast, the QI IX*—postoperative visit* and QI VII—*perioperative morbidity and mortality report* were scarcely fulfilled. Only five centers gained adherence to QI IX and only six to QI VII (Fig. 4).

### Difficulties during implementation

**Fig. 5:**
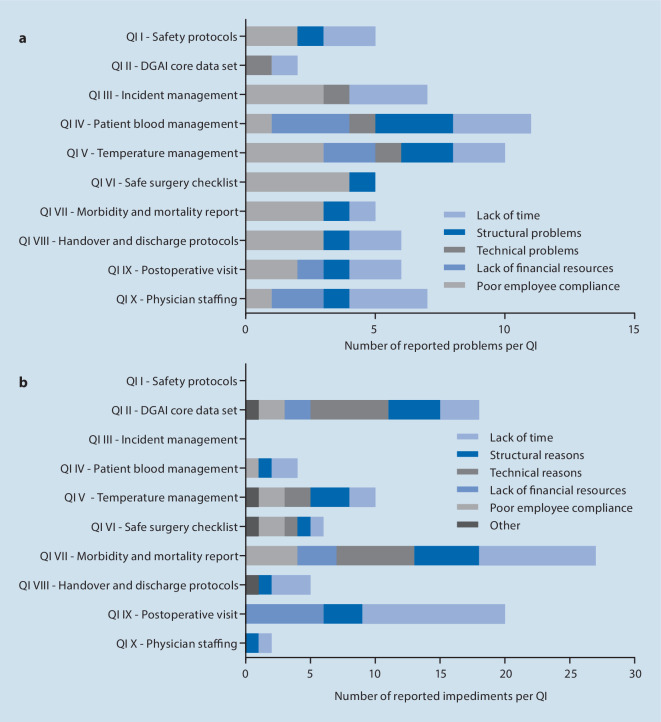
Difficulties and impediments. Difficulties during quality indicator (QI) implementation that could be solved on site (**a**) and impediments preventing implementation (**b**). Multiple answers per QI were allowed

Each of the 16 centers could theoretically adhere to a maximum of 10 points (10 QI), resulting in a maximum of 160 implementable QI in this study. At the end of the trial 109 QI were reported as implemented. For all of these successfully implemented QI, the centers were asked to report potential difficulties during the implementation phase that could be solved on site. The analysis revealed a total of 64 problems among the implemented QI. The leading problem was poor employee compliance (*n* = 22), followed by lack of time (*n* = 19), structural problems (*n* = 11) and lack of financial resources (*n* = 8) (Fig. 5a). Technical problems, such as issues concerning the PDMS, were reported four times. Only seven (43.8%) centers reported that the complete information required was available through routine data collection.

Of the 160 theoretically implementable QI 51 failed to be implemented. For those QI requiring multiple measures for implementation, the reasons for failure of implementation are listed in Table 2. Here, the centers were asked to explain reasons for non-implementation. Lack of time was reported 32 times and was the main reason impeding QI implementation during this study (Fig. 5b). Structural reasons inhibited implementation 19 times, followed by technical reasons (*n* = 15), lack of financial resources (*n* = 11), poor employee compliance (*n* = 11) and other reasons (*n* = 4). Consistently, the most frequently stated reasons inhibiting implementation were reported for the QI VII—perioperative morbidity and mortality report (*n* = 27) and the QI IX—postoperative visit (*n* = 20), which were the least implemented.

**Table 2:** Reasons for failed implementation of QI consisting of multiple criteria. Overview of the factors, leading to non-implementation of those QI, consisting of more than one criterion. Note: QI III—*incidence management* consisted of two criteria, which were implemented in all centers and is hence not shown in the table

QI	Failures (*n*)
**QI V—temperature management**	
– Documented intraoperative temperature recording in >80% of audited protocols >½ h	4
– Core body temperature >36 °C at end of surgery in >70% of audited protocols	5
– SOP temperature management available	3
**QI VI—safe surgery checklist according to the WHO**	
– WHO safe surgery checklist in patient protocol by default	0
– WHO safe surgery checklist completed in >95% of audited protocols	4
**QI VII—perioperative morbidity and mortality report**	
*Presence of item querying:*	
– Mortality (24 h postoperative)	10
– Aspiration	10
– Puncture-related lesion	8
– Awareness	9
– Patient positioning injury	10
– Visual analogue scale (VAS) >3 (when discharged from PACU)	11
– Postoperative nausea and vomiting (PONV)	9
Queried items completed in >95% of audited protocols (if available)	11
**QI VIII—handover and discharge protocols**	
– SOP/instruction for handover and discharge protocol in clinical routine	3
– Items completed in >95% of audited protocols	6
**QI X—physician staffing according to DGAI recommendations**	
– Ratio anesthetist:patient 1:1 = 100%	0
– Ratio supervisor:resident in the first 3 months of anesthesia training 1:1 or 1:2 = 100%	5

*QI* quality indicator, *SOP* standard operation procedures, *WHO* World Health Organization, *PACU* post anesthetic care unit, *DGAI* German Society of Anesthesiology and Intensive Care Medicine

## Discussion

In this trial the implementation of 10 different QI was proven to be mostly feasible. Each QI could either be implemented in at least one new center or was already implemented in the majority of the centers before. With respect to the limited resources provided within the framework of this trial, this indicates no insurmountable implementation barriers for any QI. The problems reported by the participating centers showed the need for further educational initiatives, research evidence, change in culture and financial and personnel resources for implementation of some QI.

Measuring quality of anesthesia is a challenging task. Many approaches have been made targeting mainly patient outcomes [8]. Recent work has focused on data acquired by healthcare authorities or insurances [14]. In other medical disciplines, such as intensive care medicine, national expert associations have already published comparable QI bundles. The German Interdisciplinary Association of Intensive Care and Emergency Medicine (DIVI) published a set of 10 treatment-focused QI that are already available in the third edition [11]. To the best of our knowledge, the present set of 10 QI, comprising structure, process and outcome indicators on a national level is unique in anesthesia [5].

The centers succeeded to implement new QI, even though the provided support for implementation was limited and all efforts had to be accomplished with the centers’ own resources. Nevertheless, only 2 of 10 QI (QI III and QI IV) were implemented in all 16 centres at the end of the study, indicating relevant potentials. Less than half of the participating centers adhered to the criteria of the outcome indicators QI VII—*perioperative morbidity and mortality report* and QI IX—*postoperative visit*. This carries the risk that the majority of the centers are not aware of the detailed postoperative outcome of their patients. This is an alarming result with respect to the Lancet commission on global surgery that stressed the postoperative mortality as a core indicator to improve surgical and anesthesia care that should be tracked in all hospitals worldwide by 2030 [13].

Obviously, some QI are easier to implement than others: The elaboration of a standard operation procedure (SOP, QI I), for instance, is relatively easy to accomplish in a half-year period. Other QI require specific items on the anesthesia records (QI II&VII). The adaptation of anesthesia records already established in clinical routine makes the adherence to these QI far more complex. The implementation of certain QI requires larger financial resources, which could not be provided within the framework of this trial. The QI IX—postoperative visit*,* for instance, was implemented only in five centers at the end of the study. This may be caused by the fact that for postoperative visits the attending anesthetist either needs to have a protected time after the responsibilities in the operating room or the center must provide additional personnel for this purpose. In times of increasing workload and the need of optimal capacity utilization in operating theaters, additional time for postoperative visits can hardly be allocated without additional resources. This complies with the reported reasons for the hindered implementation in the centers. Usage of the WHO safe surgery checklist, represented in QI VI, is known to reduce mortality [9]. A recent retrospective analysis showed a global completion rate of 79.1% [4]. This study showed that all 16 centers used the checklist in clinical routine and 12 of them achieved self-reported completion rates >95%, which was regarded as complete adherence to QI VI. Surprisingly, 13 centers had already fulfilled the criteria for QI VI in 2017 but 2 centers lost adherence in 2018 due to a new onset of insufficient completion rate, while only 1 center could newly implement the QI. Reasons for this can be manifold, but improvement of the employee compliance and awareness of the importance of complete safe surgery checklists might easily solve this problem. The analysis of problems during the implementation was intended to assess the need for enhanced implementation aids or other support in future. The reasons for non-implementation were questioned in order to detect potential QI, which are inappropriate due to immense implementation barriers. It must be pointed out that the differentiation between both questions was subjective and depended on the commitment of the participants. This is underlined by the fact, that 7 of the 16 centers did not implement any new QI and 1 centre even lost adherence to QI VIII in the course of the study, mostly due to lack of time. Meanwhile, the same QI could be implemented by the other participants.

The QI are usually not designed to measure a defined quality only once but to evaluate the development of quality during several reassessments. The complete adherence to all QI of a predefined set is considered to be the optimum, which can only be achieved under ideal circumstances. Therefore, it is not a deficit of the present QI if some are less implemented than others, but would allow future improvements. The German Institute for Quality Assurance and Transparency in Healthcare (IQTiG), for example, defines the potential for future improvements as a key quality criterion for the development of new QI. A QI that is implemented among all participants is not suitable for differentiation between poor and good quality. The IQTiG even recommends replacing a QI from a predefined set, if it is fully implemented during multiple re-evaluations, even if it is targeting an important issue. It is then considered to be a standard [10].

For future widespread implementation of the QI, a simple accessibility of the required data is important, e.g. data concerning perioperative morbidity and mortality should be preferably available via electronic PDMS. Only 7 of the 16 centers reported routinely recording all necessary information. The other nine centers had to acquire parts of the data manually for the purpose of this study. Thus, the adaptation of anesthesia records and PDMS systems will be an important task for hospitals willing to gain complete QI adherence in clinical routine.

It has been pointed out that feeding the gained data back to the care providers is paramount for the improvement of care [1]. Feedback reports have been shown to be an effective strategy for successful implementation of QI in studies mostly targeting the sector of internal medicine [3]. Comparative performance reports could be a next step for hospitals to estimate their individual performance, as shown by the example of performance reports among British anesthetists [2]. Subsequent to this work, it is planned to promote the broad usage of the QI among German hospitals in clinical routine. As a first step, it is planned to inaugurate a website providing benchmarking tools for hospitals willing to analyze and improve their adherence to DGAI’s QI. This medium could also be used to provide comparative feedback data to participating hospitals.

Future investigations should address whether implemented QI of anesthesia care effectively help improving patient-centered outcomes. This proof would facilitate the acceptance and dissemination of QI among anesthetic care providers.

### Limitations

This study was designed to evaluate the ability to implement the 10 anesthesia QI with limited resources. The support provided within the study framework was limited to direct implementation aids. The progress of the implementation itself depended solely on the commitment of the involved investigators. Financial or technical support could not be provided during this study to solve structural or technical barriers. An on-site monitoring was not feasible due to the limited resources. In order to disseminate the QI in further hospitals, the authors would recommend using a peer review process. Furthermore, this trial was not designed to evaluate the effect of QI implementation on the outcome of patients.

## Conclusion

Implementation of the 10 QI is mostly feasible. The implementation of the QI is a suitable tool to establish a new quality target for German anesthesia departments. Comparative data from the centers are needed for benchmarking between hospitals.
